# Grapevine *VlbZIP30* improves drought resistance by directly activating *VvNAC17* and promoting lignin biosynthesis through the regulation of three peroxidase genes

**DOI:** 10.1038/s41438-020-00372-3

**Published:** 2020-09-01

**Authors:** Mingxing Tu, Xianhang Wang, Wuchen Yin, Ya Wang, Yajuan Li, Guofeng Zhang, Zhi Li, Junyang Song, Xiping Wang

**Affiliations:** 1 State Key Laboratory of Crop Stress Biology in Arid Areas, College of Horticulture, Northwest A&F University, Yangling, 712100 Shaanxi China; 2 Key Laboratory of Horticultural Plant Biology and Germplasm Innovation in Northwest China, Ministry of Agriculture, Northwest A&F University, Yangling, 712100 Shaanxi China; 3 College of Enology, Northwest A&F University, Yangling, 712100 Shaanxi China

**Keywords:** Abiotic, Plant molecular biology

## Abstract

Drought stress severely affects grapevine quality and yield, and recent reports have revealed that lignin plays an important role in protection from drought stress. Since little is known about lignin-mediated drought resistance in grapevine, we investigated its significance. Herein, we show that *VlbZIP30* mediates drought resistance by activating the expression of lignin biosynthetic genes and increasing lignin deposition. Transgenic grapevine plants overexpressing *VlbZIP30* exhibited lignin deposition (mainly G and S monomers) in the stem secondary xylem under control conditions, which resulted from the upregulated expression of *VvPRX4* and *VvPRX72*. Overexpression of *VlbZIP30* improves drought tolerance, characterized by a reduction in the water loss rate, maintenance of an effective photosynthesis rate, and increased lignin content (mainly G monomer) in leaves under drought conditions. Electrophoretic mobility shift assay, luciferase reporter assays, and chromatin immunoprecipitation-qPCR assays indicated that *VlbZIP30* directly binds to the G-box *cis*-element in the promoters of lignin biosynthetic (*VvPRX N1*) and drought-responsive (*VvNAC17*) genes to regulate their expression. In summary, we report a novel *VlbZIP30*-mediated mechanism linking lignification and drought tolerance in grapevine. The results of this study may be of value for the development of molecular breeding strategies to produce drought-resistant fruit crops.

## Introduction

Drought is one of the most serious factors limiting agricultural productivity, and the development of drought-tolerant plants is an important objective worldwide^1–3^. Grapevine is a perennial fruit crop that is cultivated globally; however, drought stress severely affects the yield and quality of grapevine, and improvement of its drought tolerance is a high priority for the related horticultural industry.

Many studies have shown that transcription factors (TFs) are important regulators of drought stress signaling, and increasing their activity can provide plants with the ability to survive under drought conditions^1^. For example, the basic region/leucine zipper (bZIP) family has been characterized in a range of plant species, such as *Arabidopsis thaliana*^4,5^, rice (*Oryza sativa*)^6,7^, tomato (*Solanum lycopersicum*)^8^, maize (*Zea mays*)^9^, and grapevine (*Vitis vinifera*)^10,11^, and some of its members have been shown to enhance drought tolerance following overexpression in transgenic plants^5–11^.

A potentially important factor in drought tolerance in perennial woody plants is lignin, which is one of the main components of wood^12^ and contributes to many biological processes, including water conduction and mechanical support^13^. Lignin is a complex phenolic polymer derived from the phenylpropanoid pathway and is formed by the oxidative polymerization of three monolignols: coniferyl, sinapyl, and *p*-coumaryl alcohols^14^. In dicots, such as grapevine^15^, lignin polymers are composed of guaiacyl (G) units, syringyl (S) units, and low levels of *p*-hydroxyphenyl (H) units, which are synthesized from the three monolignols (coniferyl, sinapyl, and *p*-coumaryl alcohol, respectively)^14^. The key genes involved in lignin biosynthesis are *phenylalanine ammonia lyase*, *cinnamate 4-hydroxylase*, *4-coumarate-CoA ligase*, *caffeoyl CoAO-methyltransferase*, *cinnamoyl CoA reductase*, *caffeic acid O-methyltransferase*, *cinnamyl alcohol dehydrogenase*, and *peroxidase* (*PRX*)^16–19^. However, many details of the associated upstream regulatory mechanisms have yet to be elucidated.

TFs have been identified as important regulators of lignin biosynthesis in many species, such as *AtMYB46* in *A. thaliana*^20^, *PvMYB4* in switchgrass (*Panicum virgatum*)^14^, *PtMYB4* in pine (*Pinus taeda*)^21^, *EgMYB1* and *EgMYB2* in eucalyptus (*Eucalyptus grandis*)^22,23^, *PtrMYB003* and *PtrMYB021* in poplar (*Populus trichocarpa*)^24^, *ZmMYB11* in maize^25^, *OsTF1L* in rice^1^, *MdMYB88* and *MdMYB124* in apple (*Malus* × *domestica* Borkh.)^2^, and *CmMYB15* in chrysanthemum (*Chrysanthemum morifolium*)^26^. However, only a few grapevine TFs (e.g., *VvWRKY2*) regulating lignin biosynthesis have been characterized^27^, and the regulatory mechanisms are not well understood in grapevine.

The development of transcriptome sequencing and bioinformatic pipelines has facilitated the use of large-scale data mining to predict TFs involved in lignin biosynthesis. For example, based on a biclustering algorithm, Rao et al.^28^, using a comparative coexpression network analysis, predicted that seven TF families (MYB, bHLH, NAC, ERF, WRKY, C2H2, and bZIP) coordinate their activity with lignin biosynthesis genes in *A. thaliana* and switchgrass. Similarly, Quan et al.^19^ used a *cis*-regulatory motif analysis and speculated that eight TF families (MYB/SANT, bHLH, AT-Hook, TCR, TBP, HD-ZIP, C2H2, and bZIP) interact with lignin biosynthesis genes in poplar. Among these families, the regulation of lignin biosynthesis by MYB and NAC TFs has been thoroughly analyzed^29–31^, and recently, the involvement of bHLH, WRKY, ERF, and HD-ZIP family genes in wood formation was also investigated^1,28,32,33^. However, to date, little is known about how bZIP TFs are involved in lignin biosynthesis.

There is growing evidence that drought stress tolerance can be affected by lignin formation. For example, overexpression of *OsERF71* and *OsTF1L* in transgenic rice was reported to enhance lignification and drought tolerance via upregulation of lignin biosynthetic genes^1,3^. In addition, overexpression of *MdMYB88* and *MdMYB124*, two closely related genes, in apple enhanced water deficiency tolerance via the regulation of lignin deposition^2^. Thus, high levels of lignification can lead to increased drought tolerance in plants, and TFs are central regulators in this network^1,2^; however, the associated regulatory mechanisms remain largely obscure.

In a previous study, we characterized a bZIP gene from “Kyoho” grapevine (*Vitis labrusca* × *V. vinifera*), *VlbZIP30*, overexpression of which in *A. thaliana* was shown to enhance osmotic stress resistance during the seedling stages^34^. In this study, we generated *VlbZIP30-*overexpressing transgenic grapevine plants and found that they also exhibited enhanced tolerance to drought. Furthermore, *VlbZIP30* overexpression significantly increased the accumulation of lignin in the grapevine. *VlbZIP30* overexpression led to increased expression of lignin biosynthetic and drought-responsive genes through the binding of the G-box *cis*-element in the promoters of these genes, which, in turn, resulted in increased lignin deposition and improved drought tolerance. The discovery of the involvement of *VlbZIP30* in drought tolerance and a connection between lignification and drought tolerance suggests a strategy to improve grapevine drought resistance by enhancing lignin biosynthesis, which will reduce the impact of drought on fruit quality and yield.

## Materials and methods

### Plant materials and treatments

Tobacco (*Nicotiana benthamiana*) plants were grown in an illumination incubator at 25 °C under a 16-h photoperiod with a light intensity of 200 μmol m^–2^ s^–1^.

*A. thaliana* ecotype Columbia (Col-0) wild-type (WT) plants and T3 homozygous transgenic lines^34^ were grown in a growth chamber at 21 °C under a 16-h photoperiod with a light intensity of 60 μmol m^–2^ s^–1^.

Grapevine (*V. vinifera* L. cv. Thompson Seedless) seedlings were grown in the grape germplasm resource orchard of Northwest A&F University, Yangling, Shaanxi, China. The embryogenic calli of Thompson Seedless were induced from floral explants as previously described^35^. The embryogenic calli were transferred to X6 medium (Caisson, MSP24-1LT) to form proembryonal masses (PEMs), which were used for grapevine transformation. All cultures described above were maintained in the dark at 26 °C.

Rooting and vegetative propagation of WT and transgenic tissue cultures was carried out on ½MS rooting medium containing 0.2 mg l^–1^ indole-3-butyric acid and 0.02 mg l^–1^ 1-naphthylacetic acid at 25 °C under a long-day (16 h) photoperiod with a light intensity of 60 μmol m^–2^ s^–1^. The apical part (~3 cm long and with a single leaf) of an in vitro shoot was resected and transferred in a new jar for propagation of replicates. After 3 months, the transgenic and WT plantlets were transferred to plastic pots (10 × 10 × 8 cm) containing a mixture of soil (Pindstrup, Denmark) and vermiculite (1:1, v-v). After 1 month of adaptation in an illumination incubator (25 °C, 16-h photoperiod and a light intensity of 200 μmol m^–2^ s^–1^), healthy and uniformly sized plants were selected and assigned to two treatment groups. Plants in one group were subjected to drought stress by withholding water for 20 days, while plants in the other group were watered (800 mL) every 7 days and used as controls. For the experiment, six plantlets per line (transgenic lines and WT) were used as one independent experiment, and the first to third leaves from the top of the plants were sampled for RNA-seq and quantitative real-time (qRT)-PCR. The first to fourth successive internode stems from the top of the plants were sampled for qRT-PCR analysis. After freezing in liquid nitrogen, samples were stored at –80 °C. All remaining leaves were collected for analysis of relative water content (RWC), electrolyte leakage, and lignin content. All experiments were independently repeated three times.

For drought treatment of older plants, adapted 4-month-old plants were transferred to larger plastic pots (25 × 25 × 15 cm) filled with a mixture of soil and vermiculite (1:1, v-v) and grown in a glasshouse. After 4 months, the 8-month-old plants were subjected to drought stress by withholding water for 40 days, with plants watered every 10 days used as controls. For the experiment, five plants per line were used as one independent experiment, and all of the leaves were sampled for RWC and chlorophyll content analyses. All experiments were independently repeated three times.

### Generation of transgenic grapevine plants overexpressing *VlbZIP30*

**Fig. 1:**
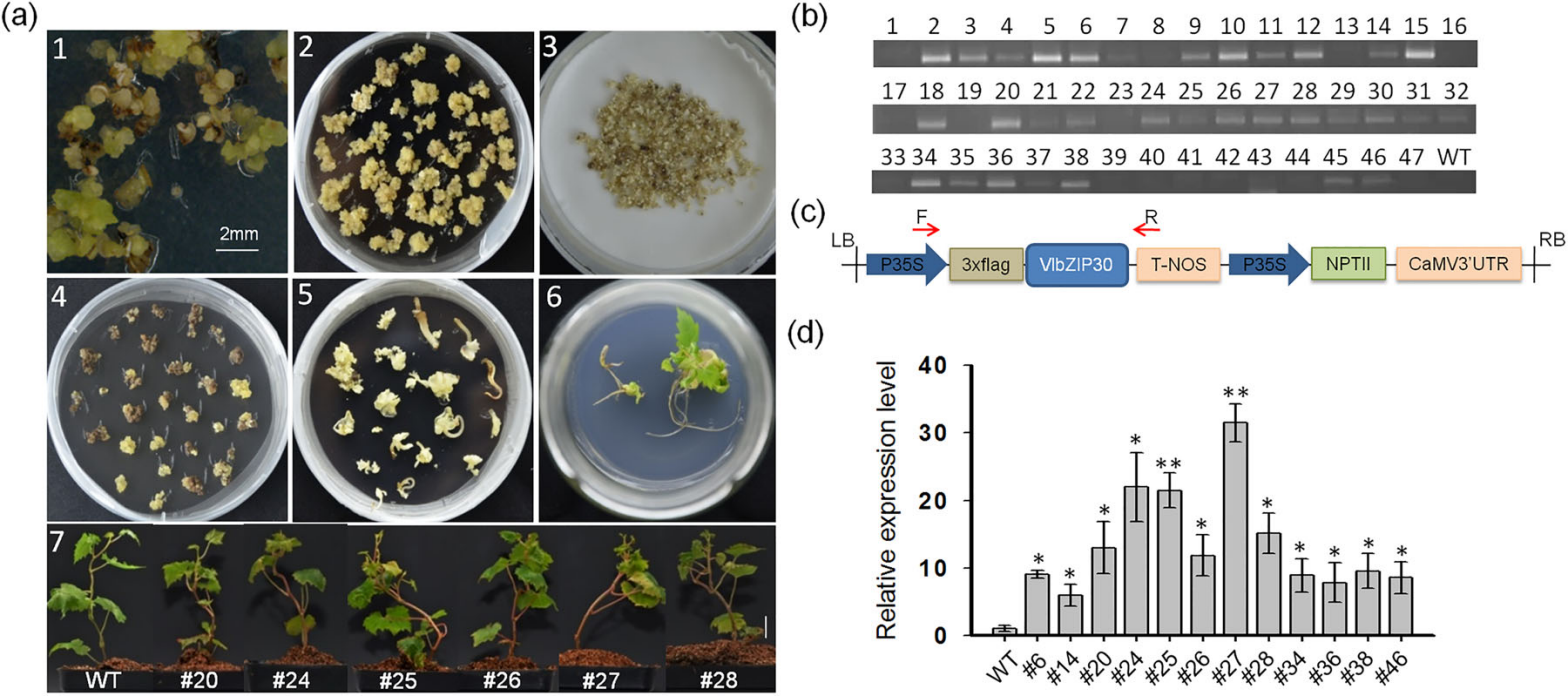
Overexpression of *VlbZIP30* in grapevine. **a** *Agrobacterium-*mediated transformation of Thompson Seedless. 1 Callus induction from flower buds. 2 Proembryonal masses (PEMs) used for transformation. 3 Coculturing of PEM and *Agrobacterium* EHA105 harboring the CaMV*35S-*3×Flag-*VlbZIP30* plasmid. 4 Kanamycin-resistant embryogenic callus. 5 Kanamycin-resistant somatic embryos. 6 Somatic embryo germination and plantlet formation. 7 Three-month-old WT and six transgenic plantlets with higher expression levels of *VlbZIP30* than the WT. **b** Identification of T-DNA insertion in 47 transgenic lines. The numbers indicate the transgenic lines. **c** Schematic diagram of the recombinant construct (CaMV*35S-*3×Flag-*VlbZIP30*). **d** Quantitative real-time (qRT)-PCR analysis of *VlbZIP30* transcript abundance in 12 transgenic lines. The grapevine *VvActin1* gene was used as an internal control. Values are means ± SEs (*n* = 3). Asterisks indicate statistical significance (*0.01 < *P* < 0.05, **P < 0.01, Student’s *t* test) between the WT and transgenic lines

The *VlbZIP30* open reading frame (ORF) was amplified and fused in frame downstream of the 3 × FLAG tag under the control of the CaMV35S constitutive promoter in the *pCAMBIA2300* expression vector using the primers presented in Table S1. The recombinant construct was verified by sequencing and renamed CaMV*35S-*3 × Flag-*VlbZIP30*. A schematic diagram of the recombinant construct is shown in Fig. 1c. CaMV*35S-*3×Flag-*VlbZIP30* was introduced into *Agrobacterium tumefaciens* strain EHA105 using the freeze-thaw method^36^ for transformation of Thompson Seedless as previously described^35^. Vector-specific primers (F: 5′-CATTTCATTTGGAGAGAACACG-3′; R: 5′-TTTGAACGATCGGGGAAAT-3′) were used to identify stable transgenic lines.

### qRT-PCR analysis

For further identification of transgenic plants, total RNA was extracted from grapevine leaves using the E.Z.N.A. Plant RNA Kit (#R6827-01, Omega Bio-tek, USA) according to the manufacturer’s protocol. For gene expression analysis, RNA was extracted from grapevine leaves and stems under both control and drought conditions using the E.Z.N.A. Plant RNA Kit. First-strand cDNA was synthesized using PrimerScript^TM^ Reverse Transcriptase (#6110A, TaKaRa) according to the manufacturer’s instructions. qRT-PCR was performed with SYBR qPCR Master Mix (#Q311-02, Vazyme) using a StepOnePlus^TM^ RT-PCR instrument (Thermo Fisher Scientific) according to the manufacturer’s protocols. *VvActin1* was used as an internal control. The gene-specific primers used for qRT-PCR are listed in Table S2. All experiments were repeated three times independently.

### Scanning electron microscopy (SEM) and histochemical analyses

Successive internodes (I1–3) were collected from the stems of 2-month-old WT and transgenic plants for SEM (Hitachi JSM-6360LV, Japan) and histochemical analyses, respectively. For each assay, five plants per line (transgenic lines and WT) were used as one independent experiment. Ten stem cross sections (from five plants) per line were used for measuring the stem diameters and xylem lengths. Internode cross sections were stained with phloroglucinol–HCl as previously described^14^. The micrographs were acquired by a Nikon DS-U3 system with a Nikon Eclipse E100 optical microscope (Nikon, Japan). Each experiment was independently repeated three times.

### Lignin extraction and quantification

4-month-old WT and transgenic plant leaves grown under both control and drought conditions and 2-month-old whole stems were collected to prepare cell wall extracts. The acetyl bromide method was used to determine total lignin content, and the thioacidolysis method was used to detect lignin composition. The H, S, and G monomers were identified and quantified by gas chromatography–mass spectrometry. All procedures were performed as previously described^14^. For each experiment, ~5 g samples (collected from ten plants) per line were considered one independent experiment, and three independent experiments were performed.

### RNA-seq and data analyses

Total RNA was extracted from 2-month-old transgenic (#25) and WT stem samples using TRIzol reagent (Invitrogen, USA), and RNA library preparation and sequencing were performed by Wuhan SeqHealth Technology Corporation (Wuhan, China). RNA-seq libraries were prepared using the KC^TM^ Stranded mRNA Library Prep Kit (#DR08402, Seqhealth, China) following the manufacturer’s instructions and sequenced using the HiSeq X10 sequencing platform (Illumina). All mapped reads were counted by featureCounts (Subread-1.5.1; Bioconductor) using annotated genes and sequences from the *V. vinifera* reference genome (http://plants.ensembl.org/index.html), and then, reads per kilobase of transcript per million mapped reads (RPKM values) were calculated. The differentially expressed genes (DEGs) between transgenic (#25) and WT plants were identified using the edgeR package (version 3.12.1), with thresholds of false discovery rate (FDR) < 0.05 and absolute log_2_FC (fold change) > 1. Kyoto Encyclopedia of Genes and Genomes (KEGG) enrichment analysis for DEGs was implemented using KOBAS software (version: 2.1.1)^37^ with a threshold of *P* value < 0.05.

For the samples of 4-month-old transgenic (#25) and WT leaves under control and drought treatments, total RNA samples were extracted using the E.Z.N.A. Plant RNA Kit (#R6827-01, Omega Bio-Tek, USA) and RNA library preparation and sequencing were performed by Biomarker (BMK) Biotechnology Corporation (Beijing, China) as previously described^34^. The DEGs between groups were identified using the edgeR package with thresholds of FDR < 0.05 and absolute log_2_FC > 1. The Venn diagrams were made using the BMK Cloud platform (www.biocloud.net). Motif predictions were performed using the promoter region 1500 bp upstream of the start codons of DEGs using DREME software (http://meme-suite.org/tools/dreme).

All sequence data in this study have been submitted to the NCBI Short Read Archive under the accession number SUB6172844.

### Evaluation of stress tolerance

Evaluation of stress tolerance Photosynthetic parameters was monitored using an LI-COR 6400 portable photosynthesis system (LI-6400XT, Huntington Beach, CA) as previously described^38^. Measurements were performed on the sixth to tenth leaves from the base on sunny days between 10:00 and 12:00 a.m. The transpiration rate, stomatal conductance, and intercellular CO_2_ concentration were analyzed using five plants per line, and three independent experiments were performed. After drought treatment, all of the leaves were collected for electrolyte leakage and chlorophyll content analyses as previously described^10^, as well as for determination of RWC^38^. The data are presented as the mean ± SD of three biological replicates.

### Electrophoretic mobility shift assay (EMSA) and dual-luciferase (LUC) reporter assay

The *VlbZIP30* coding region was amplified and cloned into the *Bam*H I and *Xho* I sites of the pGEX-6P-1 vector containing a GST tag using the primers shown in Table S1. The GST-*VlbZIP30* fusion protein was expressed in *Escherichia coli* strain BL21 (DE3) (Invitrogen) and purified using Glutathione Sepharose 4B beads (GE Healthcare, Little Chalfont, UK). EMSAs were performed using the Light Shift Chemiluminescent EMSA Kit (Thermo Scientific) as previously described^39^.

The candidate gene promoter sequences containing the ACGTG *cis*-element recognized by *VlbZIIP30* were amplified by PCR from Thompson Seedless genomic DNA (all the amplification sequences are listed in Data S1). The PCR products were ligated into the reporter vector pGreen II 0800-LUC. The full-length *VlbZIP30* ORF was amplified and cloned into the effector vector pGreen II 62-SK under the control of the CaMV35S promoter. The primers used for vector construction are listed in Table S1. The effector and reporter constructs were transformed into *A. tumefaciens* strain GV3101 (#AC1003, Weidi, Shanghai, China) containing the pSoup helper plasmid. One-month-old tobacco leaves were coinfiltrated with *A. tumefaciens* harboring the effector and different reporters as previously described^40^. Promoter activities were determined based on firefly LUC/Renilla luciferase (REN) activities using the Dual-Luciferase Reporter Assay System (Promega) with an Infinite M200 Pro microplate reader (Tecan) as previously described^41^. All experiments were independently repeated three times with similar results. For each assay, three technical replicates were performed.

### Chromatin immunoprecipitation (ChIP)-qPCR assay

For ChIP analysis, 12 g of young leaves obtained from CaMV*35S-*3×Flag-*VlbZIP30* transgenic plants grown for 4 months at 25 °C under a 16-h photoperiod and then dehydrated for 20 days were cross-linked for 15 min in 1% formaldehyde under vacuum. The ChIP assay was performed as previously described^42^ with minor modifications. The anti-FLAG M2 antibody (#F1804, Sigma-Aldrich) and the IgG (Sigma-Aldrich) control were used for immunoprecipitation. The independent transgenic line #25 and the input sample were used in the ChIP-qPCR assay. The candidate target gene primers used for ChIP-qPCR are listed in Table S2. Two independent experiments were performed with similar results. The data represent the means of three replicates ± SDs from one experiment.

### Accession numbers

Genes from this article can be found in the Ensembl Plants database (http://plants.ensembl.org/index.html) under the following accession numbers: VIT_13s0175g00120 (*VlbZIP30*), VIT_08s0058g00970 (*VvPRX1*), VIT_06s0004g07770 (*VvPRX4*), VIT_04s0023g02570 (*VvPRX72*), VIT_01s0026g02710 (*VvNAC26*), VIT_19s0014g03290 (*VvNAC17*), VIT_14s0068g00300, VIT_12s0055g01010 (*VvPRX N1*), VIT_13s0067g02360 (*VvPRX4-like*), VIT_07s0130g00220 (*VvPRX47*), VIT_04s0044g00580 (*VvActin1*). The names of these genes were obtained from the KEGG database (http://www.kegg.jp/kegg/) or published studies.

## Results

### Identification of transgenic grapevine lines overexpressing *VlbZIP30*

Previously, we characterized a bZIP gene from “Kyoho” grapevine, *VlbZIP30*, overexpression of which in *A. thaliana* was shown to enhance dehydration tolerance under mannitol treatment during the seedling stage^34^. In this study, to examine the function of *VlbZIP30* in a homologous system, the *VlbZIP30* overexpression construct was transformed into PEMs derived from Thompson Seedless (Fig. 1a). Genomic DNA was extracted from leaves of putative transgenic lines and nontransgenic control plants (WT), and 30 independent transgenic plants were confirmed by PCR (Fig. 1b). qRT-PCR was used to further assess the expression of *VlbZIP30* in transgenic lines that showed a lignified stem phenotype (Fig. 1d and Fig. S1). This phenotype was more pronounced in six lines (#20, #24, #25, #26, #27, and #28) with high expression levels of *VlbZIP30* (10- to 30-fold) than in the WT (Fig. 1). The three lines with the highest expression levels (#24, #25, and #27) were selected for further analysis.

### Overexpression of *VlbZIP30* in grapevine significantly increases lignin accumulation

**Fig. 2:**
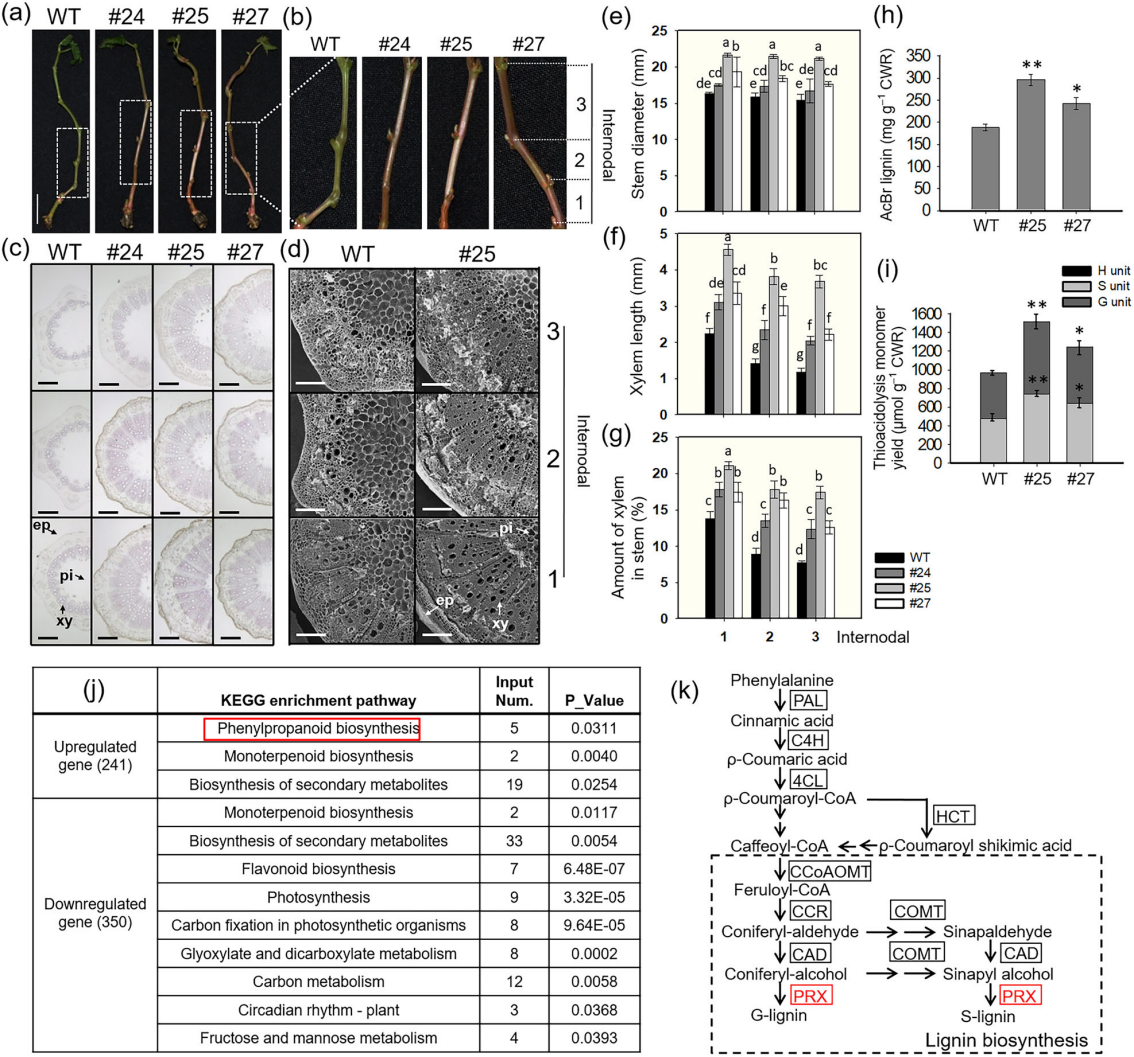
Phenotype and RNA-seq analysis of 2-month-old transgenic grapevine plants overexpressing *VlbZIP30*. **a** Visible phenotypes of Thompson Seedless (WT) and transgenic line (#24, #25, and #27) stems. Scale bar, 2 cm. **b** Successive internodes (I1–3) of WT and transgenic line stems. Phloroglucinol–HCl staining (**c**) and scanning electron micrographs (**d**) of WT and transgenic line stem cross sections (I1–3). ep epidermis, xy secondary xylem, pi pith cells. Scale bars, 200 µm. Measurement of stem diameter (**e**) and xylem length (**f**) in WT and transgenic line stem cross sections (I1–3). **g** Percentage of xylem in stems, calculated as the area of the garland structure divided by the overall stem area. Values are means ± SEs (*n* = 3). Statistically significant differences are indicated by different lowercase letters, according to Fisher’s LSD test (*P* < 0.05). **h** Total lignin content in WT and transgenic line stems. **i** Lignin composition of WT and transgenic line stems. H *p*-hydroxyphenyl unit, S syringyl unit, G guaiacyl unit, CWR cell wall residue. Values are means ± SEs (*n* = 3). Asterisks indicate statistical significance (*0.01 < *P* < 0.05, **P < 0.01, Student’s *t* test) between the WT and transgenic lines. **j** KEGG pathway enrichment analysis of upregulated and downregulated differentially expressed genes (DEGs) in the stems of a transgenic line (#25) compared with the WT. The red box represents the phenylpropanoid biosynthesis pathway. **k** A simplified schematic representation of lignin biosynthesis through the phenylpropanoid biosynthetic pathway. The red boxes represent the upregulated genes in line #25

To investigate a potential relationship between *VlbZIP30* expression and lignin biosynthesis, we examined lignin accumulation in 2-month-old transgenic plants. Morphological characterization of the transgenic lines revealed significant lignification in the stems of all the transgenic lines, especially in the basal internodes (I1–3) (Fig. 2a, b). Through SEM and phloroglucinol–HCl staining, we observed that the transgenic lines had a thicker secondary xylem than the WT in the same internodes (I1–3) of the stem cross sections (Fig. 2c, d). We also measured the stem diameter, xylem length, and percentage of xylem in the stems of the different internodes (I1–3). Statistical analyses showed that the xylem length and the percentage of xylem in the stem correlated with this phenotype (Fig. 2f, g). We found that the stems of transgenic lines increased in diameter as the degree of lignification increased (Fig. 2e).

To confirm the apparently enhanced lignin biosynthesis in the stems of the transgenic lines, we measured the amount of extractable lignin and characterized the lignin composition. We observed a 1.3- to 1.6-fold increase in total lignin in the transgenic lines (#25 and #27) compared to the WT (Fig. 2h), and the abundance of G and S units in the transgenic lines showed an increase of 1.3- to 1.5-fold and 1.2- to 1.6-fold, respectively, compared to the WT (Fig. 2i). We also imaged phloroglucinol–HCl staining in inflorescence stem cross sections of *VlbZIP30-*overexpressing *A. thaliana* lines (OE1, OE6, and OE23). However, no significant difference in staining intensity was observed compared to the WT control (Fig. S2). These results indicated that *VlbZIP30* significantly affects the accumulation of lignin in grapevine stems by increasing the synthesis of both G and S lignin monomers.

### Identification of *VlbZIP30*-regulated lignin biosynthetic genes using RNA-seq analysis

To provide further evidence supporting the connection between *VlbZIP30* and lignin biosynthesis, we performed an RNA-seq analysis of 2-month-old stems from the transgenic line (#25) and WT control plants grown under normal conditions. Two plants per line (#25 and WT) were used as one independent sample, and three independent samples were used for RNA-seq analysis. A total of 591 DEGs (241 upregulated and 350 downregulated) were expressed with at least a twofold change (FDR < 0.05) in line #25 compared to the WT, and enrichment analysis was performed for biological pathways. We identified three KEGG pathways, namely, phenylpropanoid biosynthesis (which is the primary determinant of lignin content)^19^, monoterpenoid biosynthesis, and biosynthesis of secondary metabolites, enriched among the 241 upregulated genes (Fig. 2j), while there were nine KEGG pathways enriched among the 350 downregulated genes. The nine pathways did not include the phenylpropanoid biosynthesis pathway (Fig. 2j). We identified five lignin biosynthetic genes from the phenylpropanoid biosynthesis pathway in the 241 upregulated DEGs, namely, *VvPRX1* (VIT_08s0058g00970), *VvPRX4* (VIT_06s0004g07770), *VvPRX72* (VIT_04s0023g02570), *VvCCoAOMT* (VIT_11s0016g02610), and VIT_13s0064g01720, and found that three of these genes were upregulated in the public GeneChips^®^ database of drought response genes^43^. These were *VvPRX1*, *VvPRX4*, and *VvPRX72*, which encode PRX genes involved in the final stages of lignin biosynthesis (Fig. 2k). These results suggest that *VlbZIP30* is likely to increase the accumulation of lignin by regulating the expression of these three lignin biosynthetic genes in grapevine stems.

### Overexpression of *VlbZIP30* enhances drought tolerance in grapevines

**Fig. 3:**
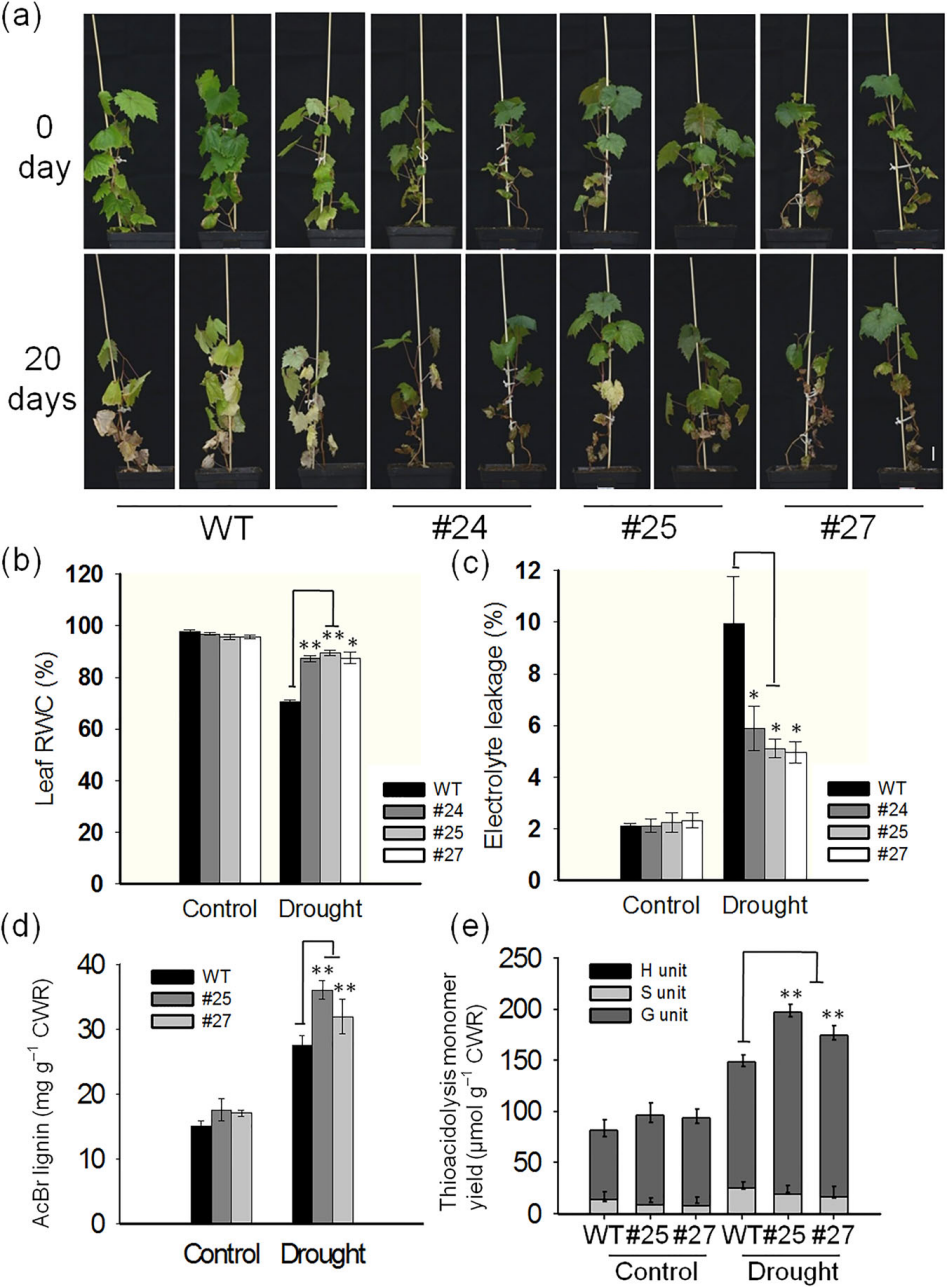
*VlbZIP30*-overexpressing grapevine plants grown in an illumination incubator show enhanced drought tolerance. **a** Drought tolerance phenotypes of Thompson Seedless (WT) and transgenic lines (#24, #25, and #27). Four-month-old plants (upper panel) were dehydrated for 20 days (lower panel). Scale bar, 2 cm. Leaf RWC (**b**) and electrolyte leakage (**c**) of WT and transgenic lines. Leaves were detached from WT and transgenic lines after both control and drought treatments. RWC relative water content. **d** Total lignin content of WT and transgenic line leaves in both the control and drought treatments. **e** Lignin composition of WT and transgenic line leaves after both control and drought treatments. H *p*-hydroxyphenyl unit, S syringyl unit, G guaiacyl unit, CWR cell wall residue. In all cases, the values are means ± SEs (*n* = 3). Asterisks indicate statistical significance (*0.01 < *P* < 0.05, **P < 0.01, Student’s *t* test) between the WT and transgenic lines

As we previously found that *VlbZIP30* overexpression in *A. thaliana* confers drought tolerance, we tested this possibility in transgenic grapevine lines (#24, #25, and #27). Four-month-old plants were selected and exposed to drought conditions for 20 days by withholding irrigation. The transgenic plants showed much less leaf wilting and yellowing than the WT plants (Fig. 3a). The degree of drought damage was assessed by standard parameters, such as RWC and electrolyte leakage^1,10,38^. Consistent with the visible phenotypes, after drought treatment, although the RWCs of all the plants decreased, they were significantly higher in the transgenic lines than in the WT (Fig. 3b). Furthermore, electrolyte leakage was significantly lower in the transgenic plants than in the WT after drought treatment (Fig. 3c). These data suggested that overexpression of *VlbZIP30* resulted in less physiological damage under drought stress in the transgenic lines than in WT plants.

Since overexpression of *VlbZIP30* in grapevine enhanced stem lignification, we investigated whether there was also increased lignification in the leaves. Under control conditions, there were no significant differences in lignin content and lignin monomer composition between WT and transgenic (#25, #27) leaves, while after drought treatment, the total lignin content of the transgenic leaves was significantly higher than that of the WT leaves (Fig. 3d). Moreover, only the lignin G monomer was significantly more abundant in transgenic leaves than in WT leaves (Fig. 3e). These results indicated that under drought stress, overexpression of *VlbZIP30* promoted the accumulation of lignin, mainly the G monomer, in grapevine leaves and enhanced the resistance to drought stress.

**Fig. 4:**
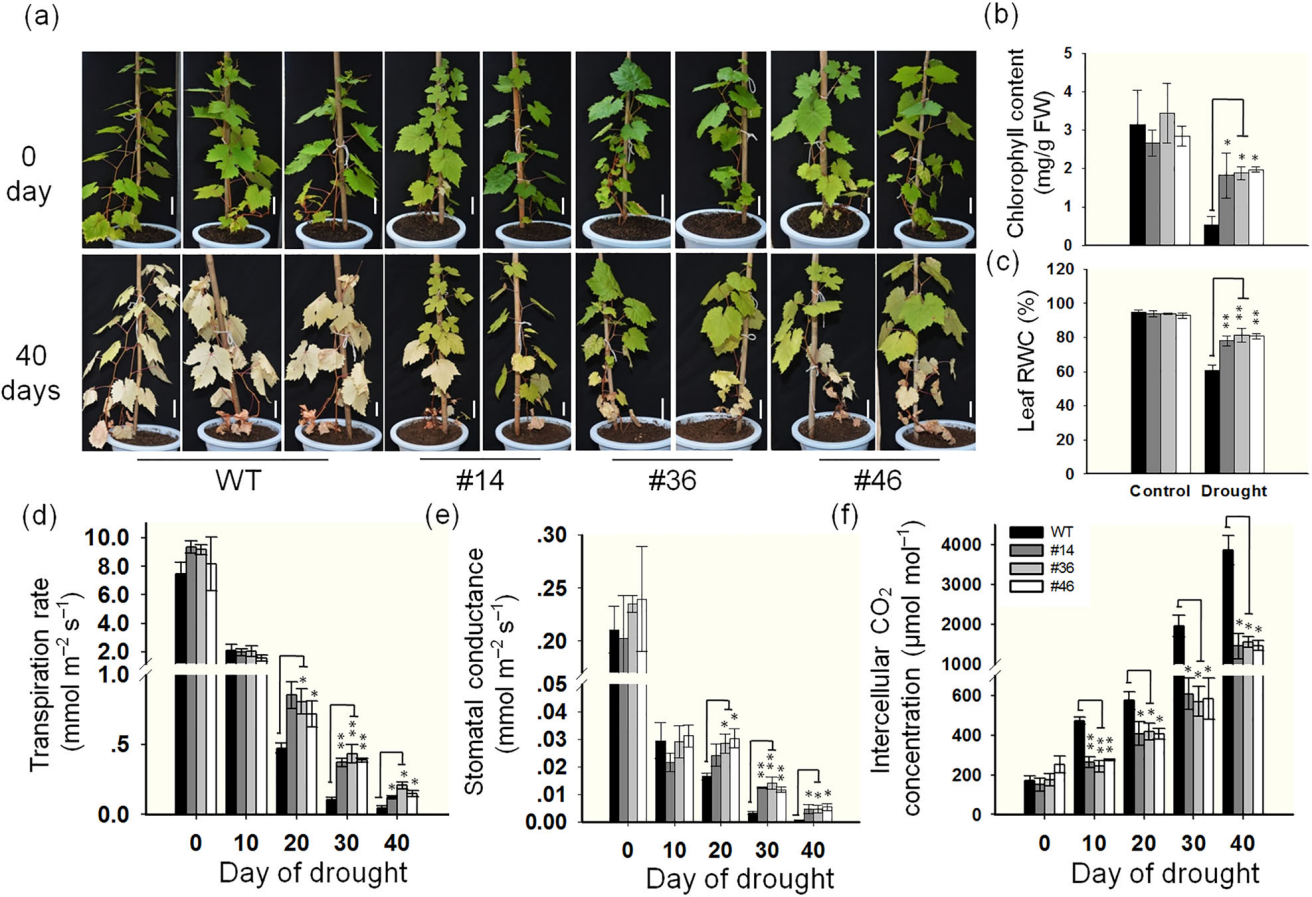
*VlbZIP30*-overexpressing grapevine plants grown under glasshouse conditions show enhanced drought tolerance. **a** Drought tolerance phenotypes of Thompson Seedless (WT) and transgenic lines (#14, #36, and #46). Eight-month-old plants (upper panel) were dehydrated for 40 days (lower panel). Bar, 5 cm. Chlorophyll content (**b**) and leaf RWC (**c**) of WT and transgenic lines. Leaves were detached from WT and transgenic lines after both control and drought treatments. RWC relative water content. Changes in photosynthetic parameters of transgenic grapevines relative to the WT during a drought period, including transpiration rate (**d**), stomatal conductance (**e**), and intercellular CO_2_ concentration (**f**). Measurements were made on sunny days between 10:00 and 12:00 a.m. In all cases, the values are means ± SEs (*n* = 3). Asterisks indicate statistical significance (*0.01 < *P* < 0.05, **P < 0.01, Student’s *t* test) between the WT and transgenic lines

To determine if the transgenic grapevines have a drought-resistant phenotype under simulated field conditions, we selected three lines (#14, #36, and #46) with *VlbZIP30* expression levels fivefold to tenfold higher than those of the WT control (Fig. 1d) for drought treatment. Eight-month-old plants were selected and exposed to drought conditions for 40 days by withholding irrigation. After the treatment, the transgenic plants showed slight leaf wilting and yellowing, while almost all the leaves of the WT were yellow and withered (Fig. 4a). Consistent with the visible phenotypes, after drought treatment, the chlorophyll content and RWC of the transgenic lines were significantly higher than those of the WT (Fig. 4b, c). The efficiency of photosynthesis is known to be directly inhibited when plants are subjected to drought stress^38^, so we evaluated photosynthesis-associated parameters in the WT and transgenic plants during drought treatment. The transpiration rates decreased sharply after 10 days from the beginning of the treatment, with no significant difference between WT and transgenic plants (Fig. 4d). However, after 20–40 days of drought treatment, transpiration rates in the transgenic lines were significantly higher than those in the WT (Fig. 4d). The stomatal conductance showed the same trend as the transpiration rate (Fig. 4e). The intercellular CO_2_ concentrations gradually increased during the drought treatment period, but with lower increments in the transgenic lines than in the WT (Fig. 4f). These data indicated that the *VlbZIP30*-overexpressing transgenic lines maintained a relatively stable photosynthetic efficiency compared to the WT control under drought conditions.

### Identification of a potential G-box motif in *VlbZIP30*-regulated drought-inducible genes using RNA-seq analysis

**Fig. 5:**
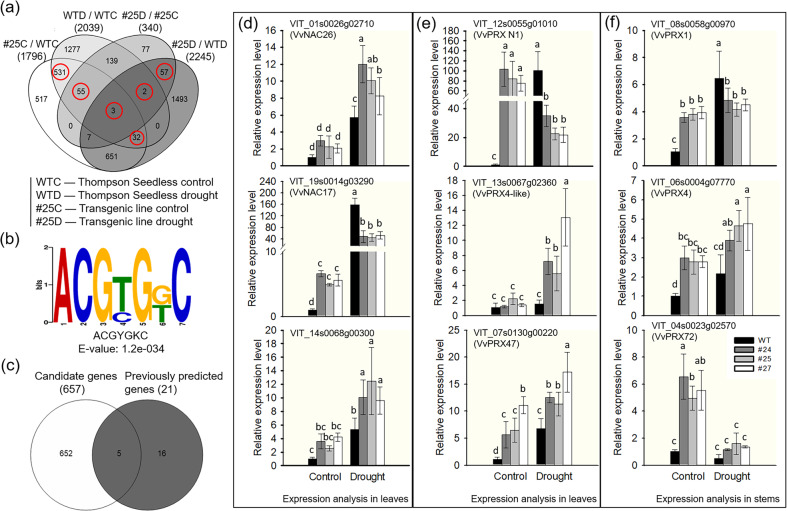
Identification of the *VlbZIP30* binding motif and VlbZIP30-regulated drought-inducible genes. **a** Venn diagram showing the number of differentially expressed genes (DEGs) in 4-month-old leaves from the transgenic grapevine line (#25) compared with Thompson Seedless (WT) under control and drought conditions for 20 days. The DEGs in red circles were selected as candidate genes for further analysis. **b** Using DREME motif analysis, a potential G-box motif sequence was found in the promoter regions of candidate genes. **c** Venn diagram of putative target genes among the candidate genes compared with the predicted 21 candidate *VlbZIP30* target genes. Quantitative real-time (qRT)-PCR analysis of drought-responsive genes (**d**) and lignin biosynthetic genes (**e**) in leaves of the *VlbZIP30-*overexpressing grapevine line (#24, #25, and #27) and WT plants under control and drought conditions. **f** qRT-PCR analysis of lignin biosynthetic genes in stems of transgenic grapevines and WT plants under control and drought conditions. The first to fourth successive internodes from the top of the plants were sampled. Values are means ± SEs (*n* = 3). Statistically significant differences are indicated by different lowercase letters according to Fisher’s LSD test (*P* < 0.05)

To examine the *VlbZIP30* regulatory network during drought stress, we performed RNA-seq analysis to identify DEGs between a transgenic line (#25) and WT plants. Four-month-old plants were exposed to drought conditions for 20 days by withholding irrigation (Fig. 3a), and leaves were sampled for RNA-seq analysis. DEGs were defined based on a threshold of twofold difference in transcript abundance (FDR < 0.05). We identified 1796 and 2245 genes that were upregulated in line #25 compared with WT plants under control (#25C/WTC) and drought (#25D/WTD) conditions, respectively (Fig. 5a). A total of 2039 and 340 upregulated genes were identified in WT (WTD/WTC) and #25 (#25D/#25C) subjected to drought stress, respectively (Fig. 5a). Subsequently, we performed KEGG enrichment analysis on the DEGs identified in the Venn diagrams (Fig. 5a) and found that the phenylpropanoid biosynthesis pathway was significantly enriched in 1796 (#25C/WTC), 340 (#25D/#25C), and 2245 (#25D/WTD) genes but not in 2039 (WTD/WTC) genes (Data S2). These results suggest that the phenylpropanoid biosynthesis pathway was significantly enhanced in transgenic lines (#25) compared to the WT under both control and drought conditions.

To better understand the role of *VlbZIP30* in drought stress signaling, the upregulated drought response genes or genes that were upregulated by overexpression of *VlbZIP30* were selected for further analyses (Fig. 5a; a total of 680 DEGs, shown in red circles). Promoter analyses were performed on these DEGs to identify candidate *VlbZIP30* target genes (removed 23 genes without a grapevine ID) using the DREME motif tool, and a potential G-box motif (Fig. 5b; ACGYGKC, E-value: 1.2e-034) was found to be significantly enriched. These 657 promoter sequences are listed in Data S3. Previous studies have shown that some bZIP proteins function as regulators of signaling networks by specifically binding the “ACGTG” G-box sequence in the promoters of their target genes and regulating their expression^9,44^. In addition, in a previous study, we hypothesized that *VlbZIP30* might participate in stress signaling in grapevine by regulating the expression of 21 grapevine genes via a putative G-box motif (MCACGTGK) in their promoters^34^. Five genes were found to be shared by the 657 candidate genes and 21 previously predicted genes (Fig. 5c). The expression of these five genes was confirmed by qRT-PCR in 4-month-old WT and transgenic plants dehydrated for 20 days, and based on these results (Figs. 5d and S3), we selected three drought-responsive genes of interest, namely, *VvNAC26* (VIT_01s0026g02710), *VvNAC17* (VIT_19s0014g03290), and VIT_14s0068g00300, for further analyses.

### *VlbZIP30* activates the transcription of drought-responsive and lignin biosynthetic genes by binding to the G-box motif in their promoters

Given that the lignin content in the leaves of the transgenic lines was higher than that in the WT under drought stress (Fig. 3d, e), we further investigated the link between lignin formation and drought stress. Among the 657 candidate genes, we identified three PRX-type lignin biosynthesis-related genes: *VvPRX N1* (VIT_12s0055g01010), *VvPRX4-like* (VIT_13s0067g02360), and *VvPRX47* (VIT_07s0130g00220). Compared with the expression in WT plants, the expression of *VvPRX4-like* and *VvPRX47* was upregulated in transgenic lines under drought stress; however, the expression of *VvPRX N1* was downregulated in transgenic lines compared with the WT under drought stress (Fig. 5e). We also examined the expression of three other lignin biosynthetic genes (*VvPRX1*, *VvPRX4*, and *VvPRX72*) selected from the stem RNA-seq data (Fig. 2j, k) and found that their expression levels were significantly higher in transgenic plants than in WT plants under control conditions (Fig. 5f), which was consistent with the RNA-seq data. Furthermore, we found that the expression of *VvPRX1* and *VvPRX4* was induced by drought stress in the WT and that, compared with the expression in WT, the expression of *VvPRX1* and *VvPRX4* was downregulated and upregulated in the transgenic plants, respectively, while the expression of *VvPRX72* did not change significantly under drought stress (Fig. 5f).

**Fig. 6:**
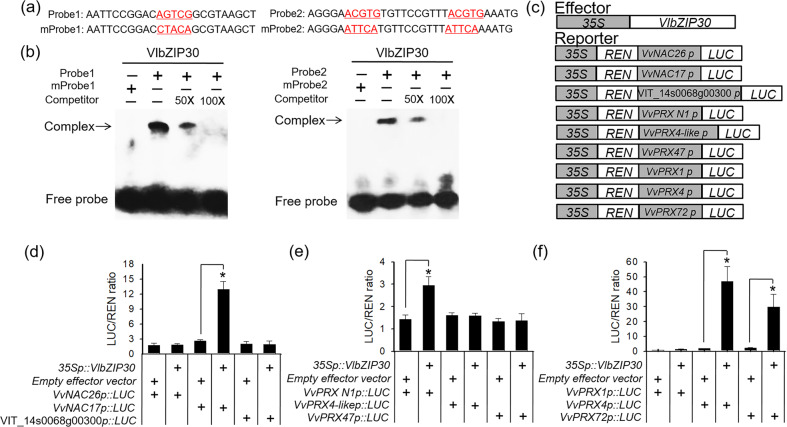
*VlbZIP30* affects the expression of drought-responsive genes and lignin biosynthetic genes by binding to G-box elements in their promoters. **a** Probes used for EMSA. **b** GST-VlbZIP30 is able to bind to fragments containing the G-box sequence from the promoter region of drought-responsive genes and lignin biosynthetic genes, as determined by EMSA analyses. Arrows indicate protein–DNA complexes. **c**–**f** Transient expression assay in tobacco (*Nicotiana benthamiana*) leaves to examine the interaction between *VlbZIP30* and the promoters of candidate target genes. **c** Schematic diagram of the effector and nine reporter constructs used for a dual-luciferase assay. Transactivation by *VlbZIP30*, shown as a ratio of LUC to REN, of the drought-responsive (**d**) and lignin biosynthetic (**e**, **f**) gene promoters. The activity of tobacco transfected with the empty vector (pGreen II 62-SK/pGreen II 0800-LUC) was set to 1. Values are means ± SEs (*n* = 3). Asterisks indicate statistical significance (*0.01 < *P* < 0.05, Student’s *t* test)

Each of the three targeted drought-responsive genes and six lignin biosynthetic genes was found to contain the G-box (ACGTG) motif in its promoter (Data S3 and S4). Based on the promoter sequences of the nine candidate target genes, we synthesized two probes containing the core binding sequence (ACGTG) and two corresponding mutant probes for EMSA (Fig. 6a) and found that the GST-VlbZIP30 fusion protein bound to both probe 1 and probe 2 (Fig. 6b) in vitro. However, the probes with the mutated G-box were not bound by this protein (Fig. 6b), further confirming the specific binding of VlbZIP30 to the G-box motif in vitro.

**Fig. 7:**
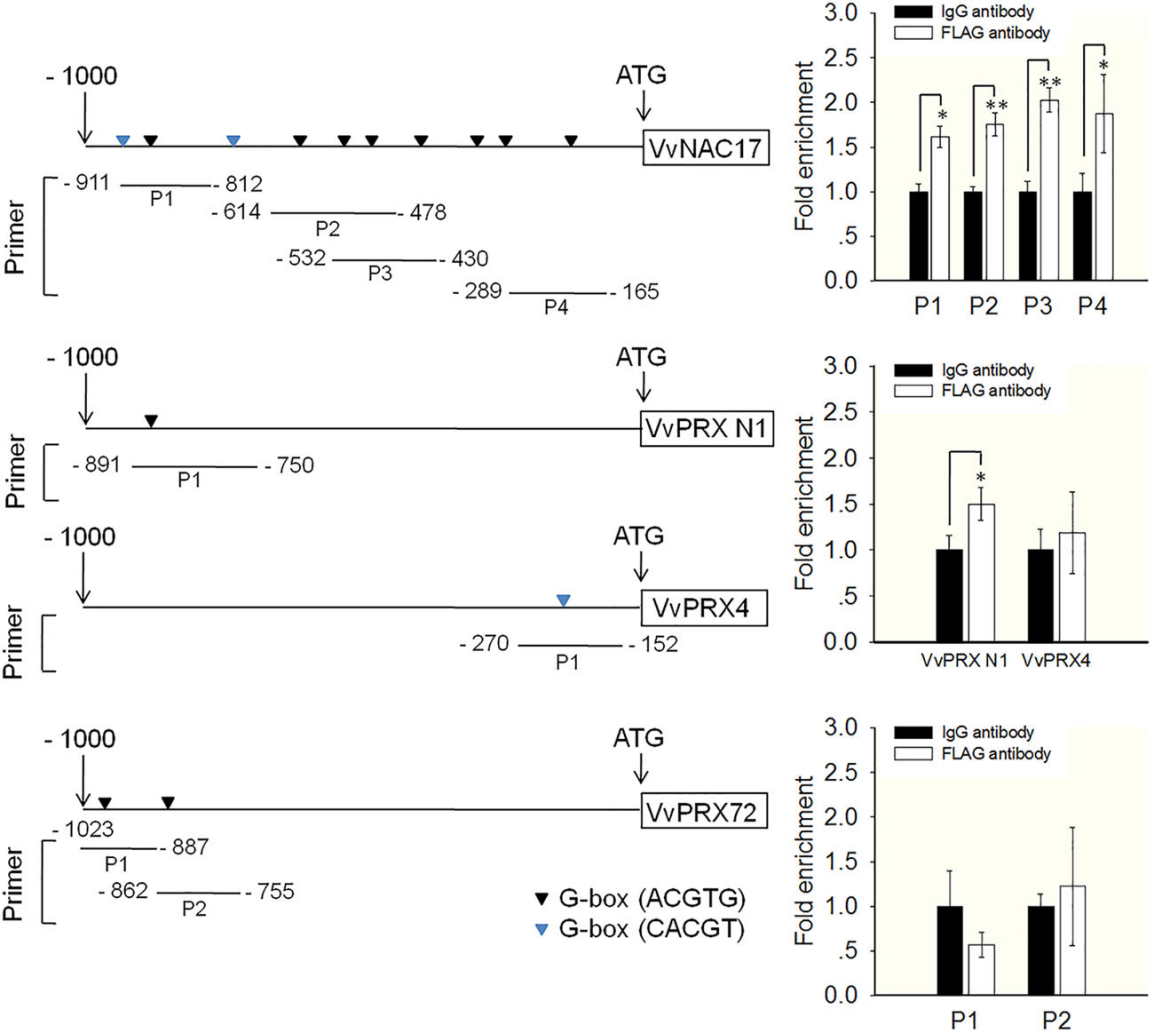
Chromatin immunoprecipitation (ChIP)-qPCR showing VlbZIP30 interacting with the promoters of four target genes involved in drought stress or lignification. Left, schematic diagram of different PCR-amplified regions used for the ChIP-qPCR assay. Right panel, relative fold enrichment of ChIP-qPCR data showing each PCR amplification region of each gene. An IgG antibody was used as a negative control. The ChIP signal was quantified as the percentage of immunoprecipitated DNA in the total input DNA, and the fold changes were calculated based on the relative enrichment in anti-Flag-*VlbZIP30* compared with anti-IgG immunoprecipitates. Data are shown as the means ± SEs of three technical replicates. Asterisks indicate statistical significance (*0.01 < *P* < 0.05, **P < 0.01, Student’s *t* test)

To further determine whether the nine candidate target genes were directly regulated by *VlbZIP30* in vivo, we performed a LUC reporter and ChIP-qPCR assay. The promoters of the nine genes were separately fused to a firefly LUC reporter sequence and cotransfected into tobacco leaves with either the 35S::*VlbZIP30* effector construct or an empty vector before the relative LUC activity was determined. A fourfold higher LUC activity was observed with the *VvNAC17* reporter, and 2- to 23-fold higher LUC activities were detected for the lignin biosynthetic gene reporters (*VvPRX N1*, *VvPRX4*, and *VvPRX72*) compared to the negative control (Fig. 6d–f). To further verify whether *VlbZIP30* can directly bind to the promoters of these four target genes, we performed a ChIP-qPCR assay with the young leaves of transgenic grapevines. As a result, the ChIP-qPCR assay using an anti-FLAG M2 antibody and a control IgG antibody showed that a significantly higher number of fragments containing the G-box of *VvNAC17* and *VvPRX N1* promoters were detected in the ChIP products than in the negative control (Fig. 7); however, no significant enrichment was detected in the ChIP products containing the *VvPRX4* and *VvPRX72* promoters than in the negative control (Fig. 7). Taken together, these results indicate that *VlbZIP30* can regulate the expression of *VvPRX4* and *VvPRX72* and that *VlbZIP30* directly binds to the *VvNAC17* and *VvPRX N1* promoters and activates their expression.

## Discussion

A large number of studies have shown that bZIP TFs play an important role in the ability of plants to resist drought stress, including in Arabidopsis^4,5^, rice^6,7^, and tomato^8^. Recent reports have revealed that lignin plays an important role in protection from drought stress. For instance, overexpression of *OsERF71* led to high lignification levels in roots via participation in cell wall modification, thereby enhancing the drought resistance of rice^3^. Overexpression of *OsTF1L* in transgenic rice was reported to enhance drought tolerance by regulating lignin accumulation and reducing the leaf RWC^1^. However, no studies have reported that bZIP TFs function in lignin biosynthesis. In this study, we showed that overexpression of *VlbZIP30* improves drought tolerance, characterized by a reduction in the leaf RWC, maintenance of an effective photosynthesis rate and increased lignin content in leaves under drought conditions. In addition, we found that *VlbZIP30* regulates the expression of lignin biosynthetic (*VvPRX N1*, *VvPRX4*, and *VvPRX72*) and drought-responsive (*VvNAC17*) genes through binding of the G-box *cis*-element in their promoters, thus promoting lignin biosynthesis and improving drought resistance in grapevine.

Previously, we identified a bZIP gene, *VlbZIP30*, overexpression of which in *A. thaliana* was shown to enhance osmotic stress resistance^34^. To further test whether *VlbZIP30* was involved in the drought stress response in a homologous system, we produced *VlbZIP30-*overexpressing transgenic grapevine plants. Twelve independent lines had lignin accumulation phenotypes in the stems (Figs. 1 and S1), suggesting that *VlbZIP30* regulates the lignin biosynthesis pathway.

To test whether *VlbZIP30* was indeed involved in regulating lignin biosynthesis, we performed SEM and phloroglucinol–HCl staining and found more lignin deposition in the secondary xylem of the transgenic lines compared to the WT control (Fig. 2). In addition, we found that overexpressing *VlbZIP30* increased the accumulation of lignin in grapevine stems mainly due to an increase in the levels of G and S units (Fig. 2h, i), which are the main components of grapevine lignin^15^. However, this regulation of lignin biosynthesis was not observed when *VlbZIP30* was overexpressed in *A. thaliana* (Fig. S2). This might be because *A. thaliana* is an herbaceous plant, while grapevines are perennial woody vines, and so these plants may differ in their lignin biosynthesis regulatory mechanisms. Based on these results, we hypothesized that *VlbZIP30* induces lignin biosynthesis in grapevine stems.

We performed RNA-seq analysis to test this hypothesis and found that genes in the phenylpropanoid biosynthesis pathway, which is critical for lignin biosynthesis^39^, were significantly enriched among the upregulated DEGs (Fig. 2j, k). We identified three putative PRX-type (*VvPRX1*, *VvPRX4*, and *VvPRX72*) target genes for *VlbZIP30*. Since some bZIP proteins modulate gene transcription by binding to a G-box (ACGTG) in the promoters of their target genes^9,44^, we performed EMSA and showed that VlbZIP30 binds to the G-box of the *VvPRX1*, *VvPRX4*, and *VvPRX72* promoters in vitro (Fig. 6b). LUC reporter assays further confirmed that *VlbZIP30* can regulate the expression of *VvPRX4* and *VvPRX72* in vivo (Fig. 6f). Furthermore, we also implemented a ChIP-qPCR assay using grapevine leaves to verify their regulatory relationships, but no significant enrichment was found. Then, we used the RNA-seq data and performed qRT-PCR analysis of leaf samples and found that *VvPRX4* and *VvPRX72* were not upregulated by *VlbZIP30* in the leaves (Fig. S4), which may be the reason for the lack of significant enrichment in the ChIP products (Fig. 7). It is speculated that in grapevine plants, *VlbZIP30* regulates the expression of *VvPRX4* and *VvPRX72* only in the stem. However, the ChIP assay remains difficult to implement in the stem.

PRX genes have been reported to be responsible for the final step in lignin formation^1,19^, and knocking out *AtPRX52* (an ortholog of *VvPRX4*) in *A. thaliana* resulted in a decrease in lignin levels due to a reduction in the expression of lignin biosynthetic genes^45^. Furthermore, knocking out *AtPRX72* (an ortholog of *VvPRX72*) in *A. thaliana* was shown to result in a decrease in lignin abundance^46^. These results suggest that *VlbZIP30* increases lignin biosynthesis in the stems of transgenic plants by regulating the expression of *VvPRX4* and *VvPRX72*.

In this study, we performed drought treatment on both 4-month-old and 8-month-old transgenic grapevines in an illumination incubator and under glasshouse conditions. Under glasshouse conditions, we found that the transgenic plants (#24, #25, and #27) with the highest expression levels of *VlbZIP30* grew slower than WT plants. This may be because these three lines use higher levels of nutrients for lignin biosynthesis in the stems than the WT, resulting in slower growth of the transgenic plants. Therefore, we selected 8-month-old transgenic grapevines (#14, #36, and #46) with *VlbZIP30* expression levels fivefold to 10-fold higher than the WT (Fig. 1d) for drought treatment. Compared with the WT, the transgenic plants showed increased leaf RWC and chlorophyll content, reduced electrolyte leakage, and an enhanced photosynthesis rate in response to drought stress (Figs. 3 and 4), factors that are all typically used as indicators of drought resistance^38^. These results suggested that *VlbZIP30* overexpression in grapevine markedly improved its tolerance to drought stress. RNA-seq analysis identified 657 candidate genes upregulated in the transgenic plants under normal or drought conditions, and a potential G-box motif was significantly enriched in the promoter regions of these genes (Fig. 5b). This result is consistent with a previous transcriptomic analysis of heterologous *VlbZIP30* expression in *A. thaliana* under osmotic stress^34^ and suggests that *VlbZIP30* participates in the drought stress response by binding to the G-box in the promoters of downstream genes.

Based on these RNA-seq data and qRT-PCR assays, we identified three drought-responsive genes (*VvNAC26*, *VvNAC17* and VIT_14s0068g00300) as candidate target genes of *VlbZIP30* (Fig. 5d). Furthermore, previous reports revealed that drought tolerance is associated with lignin formation^1,2^. To determine whether the accumulation of lignin in transgenic plants changed under drought conditions, we measured leaf lignin content and found that it was higher, mainly the G unit, in transgenic leaves than in WT leaves after drought stress (Fig. 3d, e). Subsequently, from the 657 candidate genes, we selected three lignin biosynthetic genes (*VvPRX N1*, *VvPRX4-like*, and *VvPRX47*) as candidate target genes of *VlbZIP30* and found that the expression of *VvPRX N1* and *VvPRX47* was induced by drought stress in WT plants, and the expression of *VvPRX4-like* and *VvPRX47* was induced by drought stress in the transgenic plants (Fig. 5e). Interestingly, the expression of *VvPRX N1* was significantly upregulated in the WT under drought stress, suggesting that *VvPRX N1* may play a role in the response to drought stress. Under control conditions, we found that in transgenic lines, the expression level of *VvPRX N1* was significantly higher than that in WT (Fig. 5e), suggesting that *VlbZIP30* can positively regulate the expression of *VvPRX N1*, but the lignin content was not different between the transgenic lines and the WT (Fig. 3d). However, after drought stress, the lignin content in the transgenic lines was higher than that in the WT (Fig. 3d), while the expression level of *VvPRX N1* in the transgenic lines was lower than that in the WT (Fig. 5e). These results indicate that the *VvPRX N1* gene itself cannot play a role in promoting lignin deposition. It needs to cooperate with other genes to promote lignin deposition. In addition, *VvPRX N1* may be involved in the feedback regulation mechanism in grapevine. When subjected to drought stress, the transgenic plants detected overfunctioning of the *VvPRX N1* gene and started a feedback regulation mechanism to partially suppress the expression of this gene. Interestingly, we found that the expression pattern of *VvNAC17* in transgenic plants was the same as that of *VvPRX N1* under both control and drought conditions (Fig. 5d, e). This implies that *VvNAC17* may have the same regulatory mechanism as *VvPRX N1* in transgenic plants.

Subsequently, EMSA, LUC reporter assays, and ChIP-qPCR assays indicated that *VlbZIP30* specifically binds to the G-box in the promoters of *VvNAC17* and *VvPRX N1* to regulate their expression (Figs. 6 and 7). A previous report indicated that *AtPRX71* (an ortholog of *VvPRX N1*) participates in lignification by changing the composition of lignin^47^. Furthermore, we found that overexpression of *VvABF2/bZIP39* in *A. thaliana* enhanced drought tolerance^11^ and could transiently transactivate the expression of *VvNAC17*^48,49^. Recently, it was reported that heterologous overexpression of *VvNAC17* enhanced drought tolerance in transgenic *A. thaliana*^50^. Taken together, these results suggest that *VlbZIP30* binds directly to the promoter of *VvNAC17* and regulates its expression to increase drought resistance; in addition, *VlbZIP30* also promotes lignin biosynthesis by directly activating *VvPRX N1* expression to improve the drought resistance of grapevine.

We found that *VvPRX N1*, which regulates lignin biosynthesis in leaves, is not induced by *VlbZIP30* in stems and that the *VvPRX4* and *VvPRX72* genes, which regulate lignin biosynthesis in stems, are not upregulated by *VlbZIP30* in the transgenic leaves, as determined by qRT-PCR (Fig. S4). This suggests that the lignin biosynthesis pathway is differentially regulated in different tissues.

**Fig. 8:**
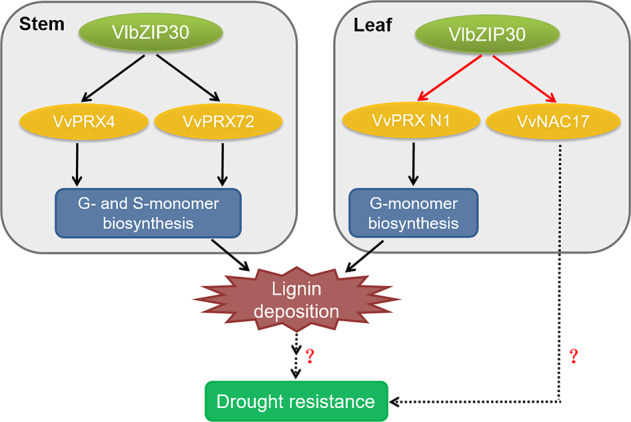
A model of the role of *VlbZIP30* in improving drought resistance in grapevine by promoting lignin biosynthesis. Solid lines and dashed lines indicate processes studied in this current study and in previous studies, respectively. Red lines indicate that *VlbZIP30* directly regulates the expression of the target genes

In a previous study, we carried out RNA-seq on *VlbZIP30*-overexpressing transgenic Arabidopsis lines under both control conditions and mannitol treatment^34^; however, no lignin biosynthetic genes were found among the DEGs, which is consistent with the phenotype in which no lignin deposition was observed in Arabidopsis (Fig. S2). This implies that the improvement of drought resistance in Arabidopsis overexpression lines is independent of lignin-related pathways. We also jointly analyzed the RNA-seq data from Arabidopsis overexpression lines and grapevine overexpression lines (Fig. 5c) and finally confirmed that overexpression of *VlbZIP30* can increase plant drought resistance by regulating *VvNAC17* (Figs. 6–8).

In conclusion, we discovered that *VlbZIP30* overexpression promotes the deposition of lignin in grapevine stems by regulating the expression of *VvPRX4* and *VvPRX72*. In addition, *VlbZIP30* directly modulates the expression of downstream genes in leaves, including lignin biosynthetic (*VvPRX N1*) and drought-responsive (*VvNAC17*) genes, which together contribute to improved drought resistance (Fig. 8). This is the first report showing that a bZIP TF is directly involved in lignin biosynthesis and enhances drought resistance in plants. The results of this study may be of value for the development of molecular breeding strategies to produce drought-resistant fruit crops.

## Supplementary information

Figure S1-4

Table S1 Primer used for vector construction

Table S2 Primers used for qRT-PCR and ChIP-qPCR

Data S1

Data S2

Data S3

Data S4
