# Supplementary material for: Grapevine VlbZIP30 improves drought resistance by directly activating VvNAC17 and promoting lignin biosynthesis through the regulation of three peroxidase genes
Source: Hortic Res. 2020 Sep 1;7:150. doi: 10.1038/s41438-020-00372-3 (PMC7458916; doi:10.1038/s41438-020-00372-3)
Supplement: Supplementary file 1 — Figure S1-4 [file 41438_2020_372_MOESM1_ESM.docx]

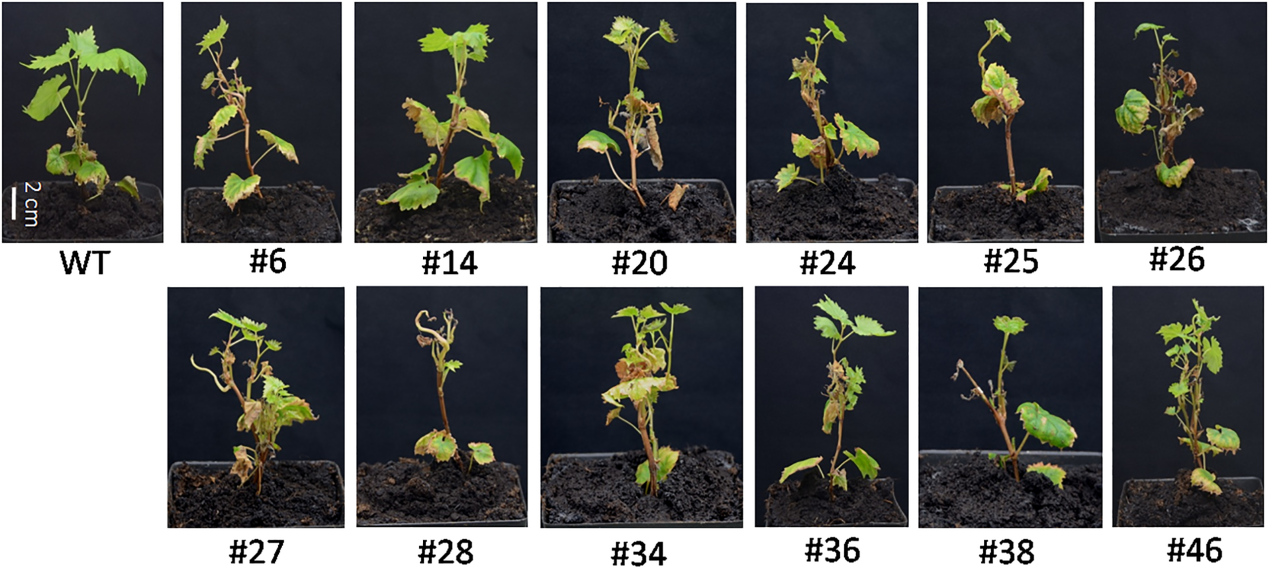


**Fig. S1** Phenotypes of Thompson Seedless (WT) and 12 transgenic lines (#6, #14, #20, #24, #25, #26, #27, #28, #34, #36, #38, #46).


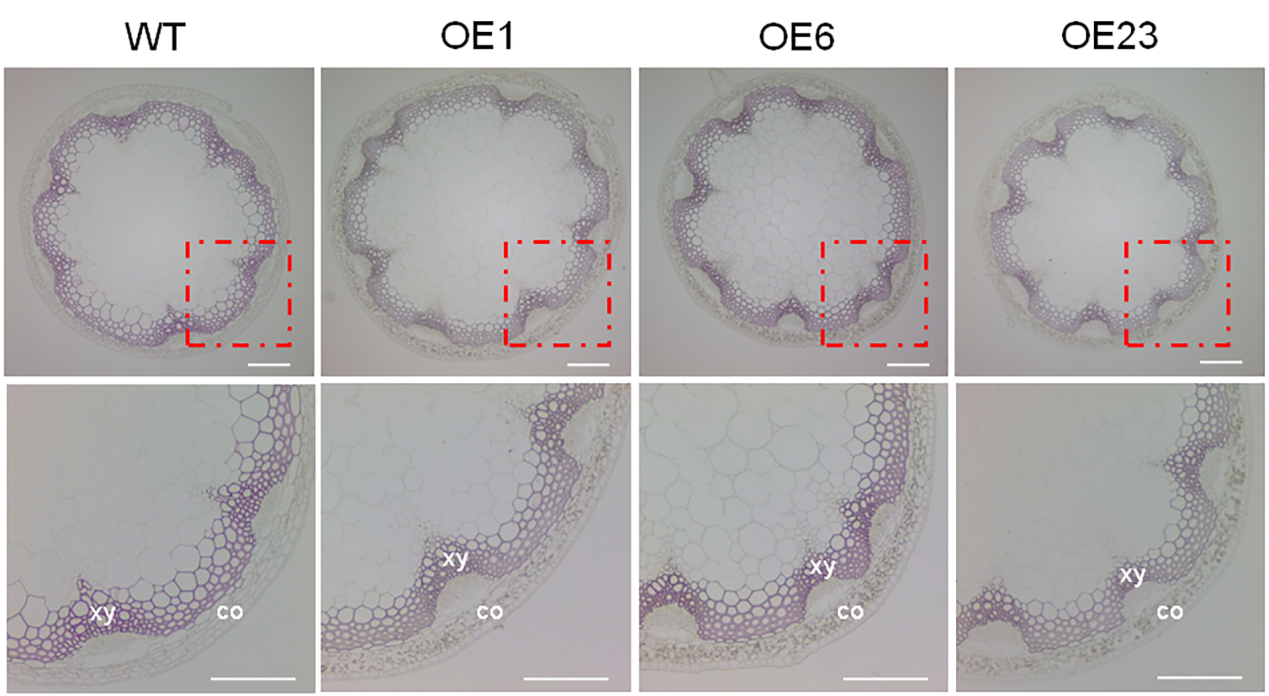


**Fig. S2** Phloroglucinol–HCl staining of inflorescence stem cross sections of 6-week-old wild type and *VlbZIP30* overexpressing *Arabidopsis thaliana*. co, cortex. xy, xylem. Scale bars, 100 µm.


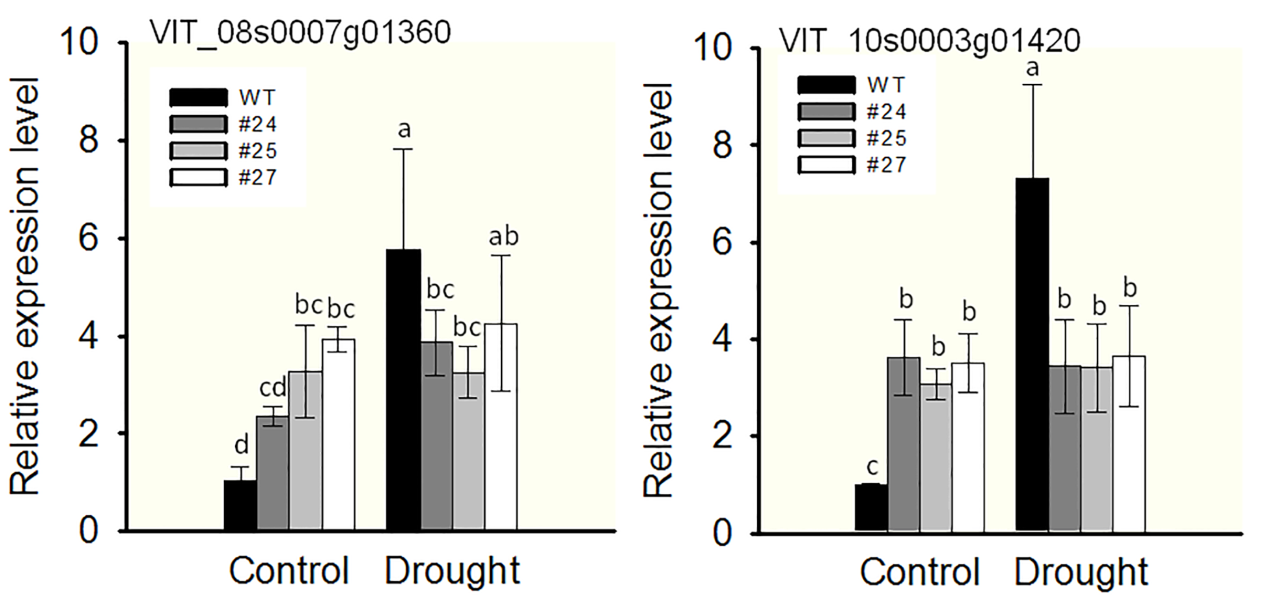


**Fig. S3** Quantitative real time (qRT)-PCR analysis of drought-inducible genes in leaves of *VlbZIP30* overexpressing transgenic grapevine lines (#24, #25 and #27) and Thompson Seedless (WT) plants under control and drought conditions. Values are means ± SE (n = 3). Statistically significant differences are indicated by different lowercase letters according to a Fisher’s LSD test (P < 0.05).


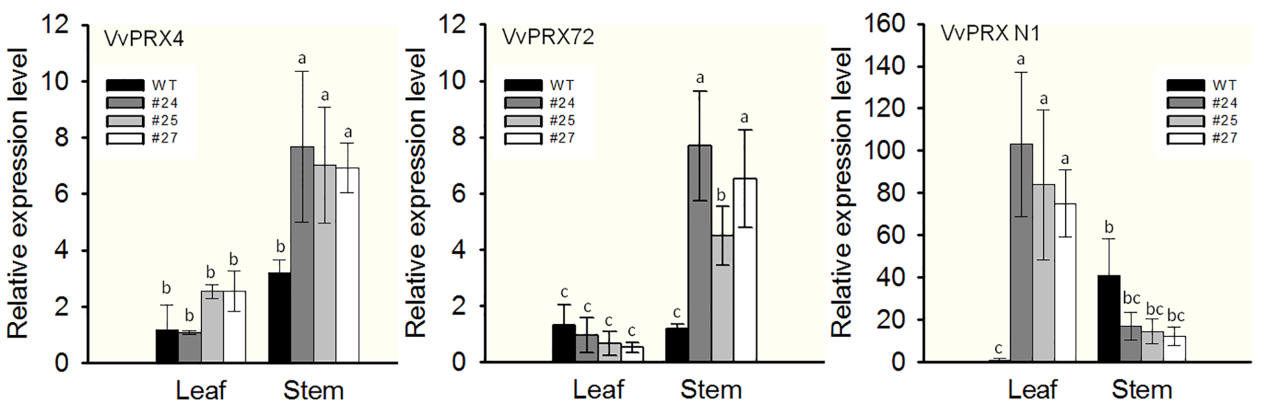


**Fig. S4** Quantitative real time (qRT)-PCR analysis of 3 target genes (*VvPRX4*, *VvPRX72* and *VvPRX N1*) involved in lignin biosynthesis in the leaves and stems of *VlbZIP30* overexpressing transgenic lines (#24, #25 and #27) under control condition, respectively. Values are means ± SE (n = 3). Statistically significant differences are indicated by different lowercase letters according to a Fisher’s LSD test (P < 0.05).
