# Supplementary material for: Grapevine VlbZIP30 improves drought resistance by directly activating VvNAC17 and promoting lignin biosynthesis through the regulation of three peroxidase genes
Source: Hortic Res. 2020 Sep 1;7:150. doi: 10.1038/s41438-020-00372-3 (PMC7458916; doi:10.1038/s41438-020-00372-3)
Supplement: Supplementary file 2 — Table S1 Primer used for vector construction [file 41438_2020_372_MOESM2_ESM.docx]

| \| Primer used for grape genetic transformation vector construction. \| \| \| \| \| \| --- \| --- \| --- \| --- \| --- \| \| Gene name \|  \| Primer sequences (5’-3’) \| \| *VlbZIP30* \|  \| f: CGCGGATCCATGGGGATTCAGACTATGGGG  R: CGAGCTCTCAGAATGGGGCTGAACTC \| |
| --- | --- | --- | --- | --- | --- | --- | --- | --- | --- | --- | --- |

F, forward; R, reverse.

| \| Primer used for vector construction of EMSA. \| \| \| \| \| \| --- \| --- \| --- \| --- \| --- \| \| Gene name \|  \| Primer sequences (5’-3’) \| \| *VlbZIP30* \|  \| f:AGTTCTGTTCCAGGGGCCCCTGGGATCCATGGGGATTCAGACTATGGGGTCTC  R:TCAGTCAGTCACGATGCGGCCGCTCGAGTCAGAATGGGGCTGAACTCGTTCTG \| |
| --- | --- | --- | --- | --- | --- | --- | --- | --- | --- | --- | --- |

F, forward; R, reverse.

| \| Specific primers used for vector construction of transactivation assay. F, forward; R, reverse. \| \| \| \| \| \| --- \| --- \| --- \| --- \| --- \| \| Gene ID \| Gene names \| Primer sequences (5’-3’) \| \| VIT_13s0175g00120  VIT_08s0058g00970  VIT_06s0004g07770  VIT_04s0023g02570  VIT_01s0026g02710  VIT_19s0014g03290  VIT_14s0068g00300  VIT_12s0055g01010  VIT_13s0067g02360  VIT_07s0130g00220 \| *VlbZIP30*  *VvPRX1*  *VvPRX4*  *VvPRX72*  *VvNAC26*  *VvNAC17*  unknown    *VvPRX N1*    *VvPRX 4-like*    *Vv**PRX47* \| f:CGCGGTGGCGGCCGCTCTAGAATGGGGATTCAGACTATGG  R:GTCGACGGTATCGATAAGCTTTCAGAATGGGGCTGAACTC  f:CTATAGGGCGAATTGGGTACCACAACTCTGTATTGACATG  R:CAGGAATTCGATATCAAGCTTGCGAGCCTCATTGTTCACT  f:CTATAGGGCGAATTGGGTACCGAAACCAAAGGAAAAAAAG  R:CAGGAATTCGATATCAAGCTTAGGACTAATCAGCCAGAAA  f:CTATAGGGCGAATTGGGTACCACCATCAGCTTATCATCCAAA  R:CAGGAATTCGATATCAAGCTTATCTTTTgTTTCCTTTTgTC  F:CTATAGGGCGAATTGGGTACCggAAATTAggCAAgCTACTgA  R:CAGGAATTCGATATCAAGCTTCGCAGCAGGTTTTCCTTTTGC  F:CTATAGGGCGAATTGGGTACCggATgCACATAAATAgTAATg  R:CAGGAATTCGATATCAAGCTTTTCCggCgATgATgATgATg  f:CTATAGGGCGAATTGGGTACCTTAATTgACAggTgATgTgA  R:CAGGAATTCGATATCAAGCTTTAATggTCTCAATTCACACA  F:CTATAGGGCGAATTGGGTACCGTGCATCAACGTGATCCTCTA  R:CAGGAATTCGATATCAAGCTTAGCAAGAGAAACAACAATGTGGG  F:CTATAGGGCGAATTGGGTACCTGAGAAACCTTTCTTTCCTT  R:CAGGAATTCGATATCAAGCTTAGAAGAGGGGGAAGCCAT  F:CTATAGGGCGAATTGGGTACCAGTGGCCAGTCAAAATAGGCA  R:CAGGAATTCGATATCAAGCTTTATTTGCCAAAGCCCAAAACCA \| |
| --- | --- | --- | --- | --- | --- | --- | --- | --- | --- | --- | --- |
