# Supplementary material for: Grapevine VlbZIP30 improves drought resistance by directly activating VvNAC17 and promoting lignin biosynthesis through the regulation of three peroxidase genes
Source: Hortic Res. 2020 Sep 1;7:150. doi: 10.1038/s41438-020-00372-3 (PMC7458916; doi:10.1038/s41438-020-00372-3)
Supplement: Supplementary file 3 — Table S2 Primers used for qRT-PCR and ChIP-qPCR [file 41438_2020_372_MOESM3_ESM.docx]

| \| Specific primers used for qRT-PCR. F, forward; R, reverse. \| \| \| \| \| \| --- \| --- \| --- \| --- \| --- \| \| Gene ID \| Gene names \| Primer sequences (5’-3’) \| \| VIT_13s0175g00120  VIT_08s0058g00970  VIT_06s0004g07770  VIT_04s0023g02570  VIT_01s0026g02710  VIT_08s0007g01360  VIT_19s0014g03290  VIT_14s0068g00300  VIT_10s0003g01420  VIT_12s0055g01010  VIT_13s0067g02360  VIT_07s0130g00220  VIT_04s0044g00580 \| *VlbZIP30*  *VvPRX1*  *VvPRX4*  *VvPRX72*  *VvNAC26*  unknown  *VvNAC17*  unknown  *VvCIPK01*  *VvPRX N1*  *VvPRX 4-like*    *Vv**PRX47*  *VvActin1* \| f: GGGGATTCAGACTATGGG  R: ATTAGCCTCAACCGTCCA  f: CCGGTGAGAAAACTGCGGTA  R: TTAAGCTCGCTGTGGTCGAG  f: ATCAGTCCTCCTCGACGACA  R: AAGCTTGACATCCCAGTCGG  f: GCTCTATCTGGGAGCCACAC  R: GGTCACCACCTGATCTTGGG  F: AATTGCCTGAGAAGGCGGAA  R: GTAGCCGGACACTGTAGCTC  F: TCCGTCTTCCACGGGTATCT  R: TAGTCCCTGGTTTGGCGTTC  F: TGAGAGTTTCACAGCCGGAC  R: TACTGGGTCGACTCTCTGGG  f: GGAAACCCAACGTGTTTCGG  R: AGTCTCTTCGCTTGCTGCTT  F: TTGCCAAGGTGGTGAGCTTT  R: ACAAGGTTAAGGTTCTGCAACA  F: AGCATGCCAAGCCTTCAGAT  R: TACCAGTGTCCAGTGCAACC  F: TGGCCATTGCTGCTAGAGAC  R: AGCTAAGGCAACCAAGTCCC  F: AACGCCTACTACATTGCGCT  R: TCAAGCAGCCCCATCTTCAG  F: GATTCTGGTGATGGTGTGAGT  R: GACAATTTCCCGTTCAGCAGT \| |
| --- | --- | --- | --- | --- | --- | --- | --- | --- | --- | --- | --- |

| \| Specific primers used for ChIP-qPCR. F, forward; R, reverse. \| \| \| \| \| \| --- \| --- \| --- \| --- \| --- \| \| Gene ID \| Gene names \| Primer sequences (5’-3’) \| \| VIT_06s0004g07770  VIT_04s0023g02570  VIT_19s0014g03290  VIT_12s0055g01010 \| *VvPRX4*  *VvPRX72*  *VvNAC17*  *VvPRX N1* \| f: ATTGCGACCGCTTCATTT  R: TTGGGATCACATTTCTACGT  f1: GGAGCCATGTACGGTCCCA  R1: GATTTCCACCCTTCATTT  F2: ACGTGGGTCCCAAGTGCA  R2: TTCGATTTAGTCTCCATCTC  F1: CATGACACGTCACAAGTCTCG  R1: GGGGAATCAATGTGGGGGTG  F2: GCTTACGTGTCGCCATTT  R2: AAGGAGGGCTTGGAGTGT  F3: ACCTCTTCGGAGGTGTCC  R3: TGGTGGTGGCGGCATTTT  F4: GTTCATGCACCGAGTGGC  R4: GTCGTCCTTGTCTCAATCTGG  F: GCATCAACGTGATCCTCT  R: TATCACCCGCTACCTACC \| |
| --- | --- | --- | --- | --- | --- | --- | --- | --- | --- | --- | --- |
